# A deep Reinforcement learning-based robust Intrusion Detection System for securing IoMT Healthcare Networks

**DOI:** 10.3389/fmed.2025.1524286

**Published:** 2025-04-08

**Authors:** Jamshed Ali Shaikh, Chengliang Wang, Muhammad Wajeeh Us Sima, Muhammad Arshad, Muhammad Owais, Dina S. M. Hassan, Reem Alkanhel, Mohammed Saleh Ali Muthanna

**Affiliations:** ^1^Department of Computer Science and Technology, Chongqing University, Chongqing, China; ^2^Department of Information Technology, College of Computer and Information Sciences, Princess Nourah bint Abdulrahman University, Riyadh, Saudi Arabia; ^3^Department of International Business Management, Tashkent State University of Economics, Tashkent, Uzbekistan

**Keywords:** Internet of Medical Things, Intrusion Detection System, CNN, LSTM, reinforcement learning

## Abstract

The Internet of Medical Things (IoMT) is transforming healthcare by enabling continuous remote patient monitoring, diagnostics, and personalized therapies. However, the widespread deployment of these devices introduces significant security vulnerabilities due to limited resources and inadequate network protocols. Intrusions within IoMT networks can compromise patient privacy, disrupt critical medical services, and jeopardize patient safety. To address these challenges, we propose HCLR-IDS, an advanced Intrusion Detection System (IDS) specifically designed for IoMT networks. The system integrates Convolutional Neural Networks (CNN), Long Short-Term Memory (LSTM) networks, and Reinforcement Learning (RL) techniques, namely Deep Q-Network (DQN) and Proximal Policy Optimization (PPO), to enhance the detection of evolving threats. The methodology begins with Enhanced Mutual Information Feature Selection (MIFS) to preprocess the CICIoMT2024 dataset, selecting the most relevant features while reducing noise and computational complexity. These selected features are then passed through a hybrid CNN-LSTM architecture. The CNN captures spatial patterns in network traffic, while the LSTM identifies temporal patterns. This dual feature extraction approach enables the system to effectively detect both static and dynamic characteristics of IoMT data. After feature extraction, the model incorporates DQN and PPO for decision-making. DQN optimizes actions based on Q-values, enhancing detection rewards, while PPO ensures stability in dynamic environments through a clipping mechanism. This combination of adaptive Q-learning and stable policy optimization significantly improves system robustness, ensuring effective real-time intrusion detection. The model demonstrates exceptional performance with binary classification accuracy of 0.9958, outperforming traditional IDS models. Additionally, it performs effectively in multi-class classification across 18 classes, achieving an accuracy of 0.7773. These results highlight that HCLR-IDS offers a reliable and efficient solution for securing IoMT healthcare systems.

## 1 Introduction

The Internet of Things (IoT) has revolutionized numerous industries, and in healthcare, it has paved the way for the Internet of Medical Things (IoMT), which connects medical devices, wearables, and sensors to improve patient care, enhance diagnostics, and enable real-time monitoring (1–3). The integration of IoMT in healthcare enables continuous patient health monitoring, predictive health management, and personalized data-driven medical interventions. These advancements are particularly impactful for patients with chronic conditions, providing opportunities for timely, preventative care and reducing the burden on healthcare systems (4). However, the rapid adoption of IoMT devices introduces critical security and privacy challenges, such as unauthorized access, data breaches, and cyberattacks, which jeopardize patient safety, confidentiality, and the integrity of medical services (5–7).

One of the most pressing challenges in securing IoMT networks is the inadequacy of traditional Intrusion Detection Systems (IDS) to address the unique characteristics of these environments. Existing IDS schemes face several limitations (8–10), including their inability to effectively capture both spatial and temporal patterns in network traffic (11), resulting in missed detections or high false positive rates (12). In IoMT networks, medical devices and sensors generate data streams that exhibit both spatial and temporal dependencies spatial patterns reflect device communication behaviors, while temporal patterns capture the evolution of attack events over time. Most existing IDS solutions, however, focus only on either spatial or temporal features, failing to capture the complex interactions between them (13). This inability to detect the dynamic and evolving nature of attacks in IoMT networks makes traditional IDS approaches unsuitable for securing such systems (14).

Moreover, the high false positive rates in traditional anomaly-based IDS systems, particularly in IoMT environments, arise from the dynamic and diverse nature of normal traffic. The communication patterns between devices can fluctuate based on patient conditions, device configurations, and environmental factors, making it difficult for conventional IDS to distinguish between benign and malicious activities. This problem is exacerbated in real-time monitoring scenarios, where delays or inaccuracies in detection can have serious consequences for patient care (15). Another significant challenge is the evolving nature of cyberattacks targeting healthcare systems. Attackers continuously develop new strategies, exploiting vulnerabilities in IoMT devices and networks (16). Traditional IDS models that rely on static detection algorithms are ill-equipped to adapt to new and emerging threats. Consequently, these models often fail to detect sophisticated attack vectors, compromising the overall resilience of IoMT security (17).

To address these pressing challenges, we propose HCLR-IDS, an advanced Intrusion Detection System specifically designed for IoMT networks. HCLR-IDS integrates state-of-the-art CNN and LSTM networks, and RL techniques to overcome the limitations of existing IDS solutions. This hybrid approach is tailored to the unique characteristics of IoMT traffic, enabling the system to capture both spatial and temporal patterns simultaneously, while integrating and adapting to evolving attack strategies. The first key feature of HCLR-IDS is the use of MIFS to preprocess the CICIoMT2024 dataset. MIFS identifies the most relevant features, reducing noise and computational complexity, and ensuring that only the most critical data is used for model training and detection.

Next, we leverage the strengths of CNNs and LSTMs to capture spatial and temporal dependencies in IoMT network traffic. CNNs are effective at detecting spatial patterns, such as device behaviors, while LSTMs are designed to identify temporal patterns, such as the sequence of events over time. By integrating these two powerful architectures, HCLR-IDS is able to detect complex, dynamic attack patterns that evolve over time an ability that traditional IDS solutions often lack. Finally, we integrate two RL models: DQN and PPO. DQN optimizes actions based on Q-values, maximizing detection rewards, while PPO enhances policy learning through a clipping mechanism, stabilizing decision-making in dynamic environments. This combination of RL techniques allows HCLR-IDS to continuously adapt to new attack scenarios and optimize its detection strategy over time, improving accuracy and reducing false positives.

The main contributions of this work include.

- HCLR-IDS integrates deep learning for feature extraction and reinforcement learning for decision-making, enabling real-time detection and adaptation to evolving threats in resource-constrained IoMT environments.
- We utilize Enhanced Mutual Information Feature Selection (MIFS) for feature selection improves efficiency by reducing noise and improving detection accuracy.
- We propose a novel hybrid framework combining Convolutional Neural Networks (CNN) and Long Short-Term Memory (LSTM) to capture both spatial and temporal patterns in IoMT network traffic. This integration addresses the challenge of dynamic and evolving attack patterns specific to IoMT environments.
- The integration of Deep Q-Networks (DQN) and Proximal Policy Optimization (PPO) enables our system to adapt and learn from real-time data, improving detection accuracy and reducing false positives. This is a critical improvement over traditional IDS approaches, which lack such adaptive capabilities.

The paper is organized as follows: Section 2 presents a comprehensive literature review, discussing relevant research in the field. Section 3 details our proposed approach for securing IoMT networks, including a description of the algorithm and its implementation. Section 4 outlines the experimental setup used in our simulations. Section 5 discusses the results, comparing the performance of our approach with existing methods. Finally, Section 6 concludes the paper and provides directions for future research.

## 2 Literature review

Intrusion Detection for the IoMT healthcare systems have attracted significant attention due to the increasing reliance on connected medical devices in healthcare environments, which exposes systems to various cybersecurity risks. As IoMT networks are becoming more widespread, addressing these emerging threats is critical for ensuring the safety and integrity of healthcare systems. Several studies have explored IDS enhancements to tackle these challenges effectively. Anomaly-based IDS is widely adopted in IoMT networks due to its ability to detect deviations from normal network behavior, which is essential for identifying novel attacks that traditional signature-based methods may miss. However, anomaly-based systems face significant challenges in dealing with false positives and capturing both temporal and spatial dependencies inherent in IoMT traffic. The traffic patterns in IoMT environments are often device-specific and highly variable, making it difficult to identify deviations without robust models capable of learning these complex behaviors (18). This limitation highlights the need for advanced methods capable of handling these complexities and providing reliable detection in dynamic environments.

Feature selection plays a crucial role in improving the performance of IDS by reducing data dimensionality and enhancing classification efficiency. Recent studies have shown the effectiveness of Improved MIFS in enhancing detection accuracy, particularly in cases where attack patterns are underrepresented or difficult to identify (19). Furthermore, hybrid approaches, such as combining Principal Component Analysis (PCA) with optimization algorithms like Grey Wolf Optimization (GWO), have been explored to optimize feature selection in deep learning-based IDS models, further enhancing performance in IoMT settings (20). These techniques demonstrate the potential to reduce feature redundancy and improve model interpretability. A meta-learning techniques are gaining traction for adapting IDS systems to evolving attack strategies. Meta-learning enables IDS models to dynamically adjust their learning strategies based on incoming network data, thereby ensuring that the detection system remains robust in the face of emerging attack patterns (21). The integration of swarm intelligence with neural networks has also been investigated to optimize decision-making processes, which can improve detection accuracy and minimize false positives in IoMT environments (22).

In addition, LSTM networks have gained prominence due to their ability to capture complex temporal patterns in network traffic, which is crucial for real-time intrusion detection. Recent studies have demonstrated that when enhanced with cyber twin deep learning techniques, LSTMs can handle the intricate temporal dependencies characteristic of IoMT traffic, leading to more accurate intrusion detection in dynamic and resource-constrained environments (23). This capability is critical for ensuring that IDS can adapt to the evolving nature of network behavior and detect previously unseen attacks. With respect to privacy-preserving approaches, federated learning has emerged as a promising solution. This method enables multiple IoMT devices to collaboratively train IDS models without sharing sensitive data, addressing privacy concerns while maintaining model accuracy. Federated learning has demonstrated its potential to improve the robustness and scalability of IDS systems in healthcare networks (24). Furthermore, hierarchical federated learning has been proposed to further enhance scalability and efficiency, making it better suited for large-scale IoMT deployments while ensuring data privacy (25).

Furthermore, in the context of Reinforcement Learning (RL), recent advancements have shown that DQN and PPO can significantly enhance decision-making and adaptability in IDS. DQN’s trial-and-error learning mechanism and PPO’s stable policy optimization have been effectively employed to improve IDS performance over time by adapting to new and emerging attack patterns. Specifically, RL-DQN models utilize a Markov Decision Process (MDP) to optimize decision-making and improve classification accuracy by learning from observed network behaviors (26–29). These techniques contribute to the overall resilience of IDS systems by enabling them to continuously evolve and respond to new threats. Table 1 describes each abbreviation and its corresponding definition.

**Table 1:** List of abbreviations.

Abbreviation	Definition
IoMT	Internet of Medical Things
IDS	Intrusion Detection System
CNN-LSTM	Convolutional Neural Networks-Long Short-Term Memory
HCLR-IDS	Hybrid CNN-LSTM Reinforcement Learning-based Intrusion Detection System
MIFS	Mutual Information Feature Selection
CNN	Convolutional Neural Networks
LSTM	Long Short-Term Memory
RL	Reinforcement Learning
DQN	Deep Q-Network
PPO	Proximal Policy Optimization
IoT	Internet of Things
IIoT	Industrial Internet of Things
MDP	Markov Decision Process
ROC	Receiver Operating Characteristic
AUC	Area Under the Curve

## 3 Proposed HCLR-IDS

**Figure 1:**
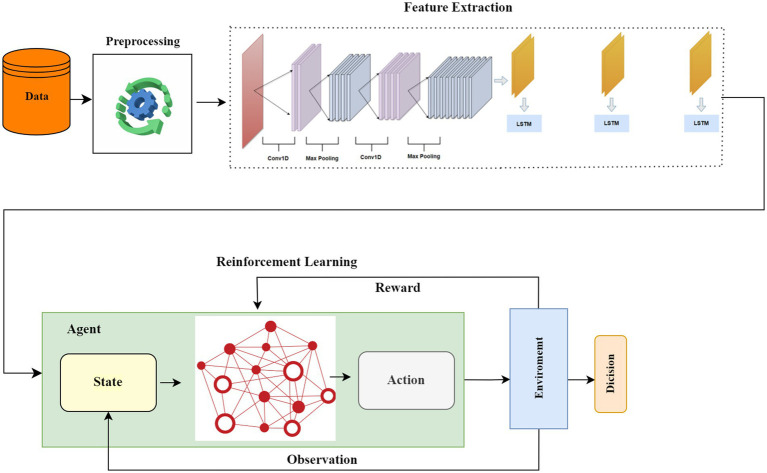
Proposed HCLR-IDS architecture.

In this study, we propose HCLR-IDS, a novel IDS for IoMT healthcare networks. The system integrates networks, and RL techniques, specifically DQN and PPO, to effectively address the complexities of IoMT traffic. The methodology begins with the use of MIFS for preprocessing the CICIoMT2024 dataset, which reduces noise, improves computational efficiency, and ensures that only the most relevant features are used for model training. The model then leverages the hybrid architecture of CNNs for spatial feature extraction and LSTMs for capturing temporal dependencies, enabling it to detect both spatial and temporal patterns in IoMT traffic. This hybrid feature extraction significantly enhances the ability to identify evolving attack patterns that traditional IDS solutions may miss. For decision-making, DQN optimizes actions based on Q-values, enhancing the detection rewards, while PPO stabilizes the learning process with a clipping mechanism, ensuring consistent performance in dynamic attack environments. This integration of CNN, LSTM, and RL enables HCLR-IDS to continuously learn from new data, adapt to emerging threats, and reduce false positives, making it a robust and real-time solution for intrusion detection in IoMT healthcare networks. Figure 1 illustrates the working principle of the proposed HCLR-IDS model.

### 3.1 Dataset and preprocessing

The CICIoMT2024 (30) dataset, utilized in this study, comprises network traffic data from 40 IoMT devices, including 25 real devices and 15 simulated devices, commonly found in healthcare environments. The dataset includes 18 distinct cyberattack scenarios, categorized into five major attack types: DDoS (Distributed Denial of Service), DoS (Denial of Service), Reconnaissance, MQTT-based attacks, and Spoofing, with a total of 3.2 million data points. These attacks include SYN Flood, ICMP Flood, UDP Flood, Ping Sweep, Vulnerability Scan, and ARP Spoofing, among others. The dataset also contains benign traffic to ensure a balanced representation of both malicious and non-malicious activities given in Table 2. To mitigate the potential impact of class imbalance on model performance, we employed stratified sampling during the data splitting process, ensuring that each subset (training, validation, and test) contained a representative distribution of attack types. This approach guarantees that the model is exposed to all attack types during training, improving its ability to generalize to unseen attack scenarios. Additionally, techniques such as oversampling and class weighting were used to further address any class imbalances during the model training phase. The data was collected using a network tap, capturing real-time packets from IoMT devices under various operational states (power, idle, active, and interaction states), reflecting real-world behaviors and threat vectors. Mutual Information Feature Selection (MIFS) was applied to reduce dimensionality, selecting 34 features with the highest mutual information to retain the most informative attributes for intrusion detection.

**Table 2:** Quantification display of the utilized dataset.

lass category	Attack type	Count
BENIGN	–	230,339
DoS	DoS TCP	462,480
	DoS UDP	704,503
	DoS SYN	540,498
DDoS	DDoS TCP	987,063
	DDoS UDP	1,998,026
	DDoS ICMP	1,887,175
Reconnaissance	Port Scan	106,603
	OS Scan	20,666
Spoofing	ARP Spoofing	17,791
MQTT	MQTT DoS Connect Flood	15,904
	MQTT DoS Publish Flood	52,881
	MQTT Malformed Data	6,877
ATTACK	–	6,800,467

*Preprocessing Steps*: The following preprocessing steps were applied to ensure that the raw network traffic data was appropriately prepared for training an effective intrusion detection model.

- *Handling Missing Values*: The dataset contained some missing values, which were addressed using two primary strategies:

- *Imputation*: Missing values were estimated using standard imputation techniques, such as mean imputation for continuous features and mode imputation for categorical features. This method ensures that valuable data points are retained without introducing significant bias.
- *Elimination*: Entries with a high percentage of missing values (beyond a predefined threshold) were removed to prevent the model from being misled by incomplete data.

To address missing and anomalous data points, we implemented a combination of imputation and elimination strategies. For continuous features, we used mean imputation to replace missing values, ensuring that the overall distribution of the data remained consistent. For categorical features, mode imputation was used to fill in missing values with the most frequent category. When missing values exceeded 20% for any entry, we eliminated that entry to avoid introducing significant bias into the dataset. These steps ensured that the dataset remained representative and suitable for training the IDS while minimizing the impact of missing or anomalous data.

- *Feature Scaling*: To standardize the dataset and ensure consistent treatment of features with different ranges, min-max scaling was employed to transform the feature values into a range between 0 and 1. The formula for min-max scaling is as follows.

(1) $$F\left(p,q\right)=\frac{F\left(p,q\right)-\min\left(F\left(p,q\right)\right)}{\max\left(F\left(p,1\right)\right)-\min\left(F\left(p,q\right)\right)}$$

Where $F\left(p,q\right)$ represents the value of the feature at row p and column q . This scaling ensures that each feature contributes equally to the model, especially in algorithms that are sensitive to the scale of data, such as distance-based models. While min-max scaling improves uniformity across features, it is sensitive to outliers. Features with extreme values could disproportionately influence the scaling process, leading to skewed results. Future work could consider using more robust scaling techniques, such as Z-score normalization or robust scaling, which are less affected by outliers.

- *Categorical Feature Encoding:* Categorical features, such as protocol types and device identifiers, were encoded into numerical values using standard methods like one-hot encoding and label encoding. These encoding techniques are necessary for enabling machine learning models to process categorical data, as most models require numerical input.
  One-hot encoding can significantly increase the dimensionality of the dataset, especially for features with a high number of unique categories. This increase in dimensionality can lead to sparsity in the feature space, making the model more complex and computationally expensive. Future work could explore alternative encoding methods, such as target encoding or embedding layers, to address these challenges and reduce the dimensionality.
- *Enhanced MIFS:* The feature selection process was conducted using the Enhanced MIFS technique to identify the most relevant features for intrusion detection (31). Initially, the dataset consisted of 42 features, and MIFS was applied to evaluate the mutual information between each feature and the target variable (i.e., the attack classes). A threshold of 0.1 for mutual information was set, allowing only features with a score above this threshold to be retained. This process reduced the feature set from 42 to 34, which significantly enhanced the model’s computational efficiency while maintaining the key information necessary for accurate attack classification. Features with minimal correlation to the attack types, such as certain packet-level attributes, were discarded, ensuring that the model focused on the most informative features. MIFS works by measuring the mutual information between features and the target variable, helping reduce dimensionality while preserving relevant information. The mutual information (MI) between two discrete variables is calculated using the formula.

(2) $$MI\left(X;Y\right)=H\left(X\right)-H\left(X|Y\right)=\underset{y\in Y}{\sum }\underset{x\in X}{\sum }p\left(x,y\right)\log\frac{p\left(x,y\right)}{p\left(x\right)p\left(y\right)}$$

Where *H*(*X*) represents the entropy of *X*, and *H*(*X*∣*Y*) represents the conditional entropy of XXX given YYY. The joint distribution $p\left(x,y\right)$ and the marginal distributions $p\left(x\right)\;\mathrm{and}\;p\left(y\right)$ are used to quantify the relationships between the variables and assess the amount of information shared between them. Although MIFS is effective for feature selection, it assumes conditional independence between features, which may not always hold in complex real-world datasets such as IoMT traffic, where feature interactions are common. Additionally, MIFS may become less effective in high-dimensional datasets, as it might overlook subtle but crucial feature relationships.

### 3.2 Proposed CNN-LSTM neural networks

**Figure 2:**
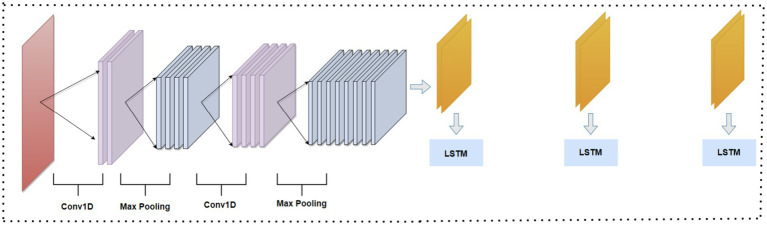
Proposed CNN-LSTM integration architecture.

This paper presents a hybrid model that combines CNN and LSTM networks for feature extraction, consisting of a convolutional layer followed by an LSTM layer, as illustrated in Figure 2. The CNN effectively extracts significant spatial features from the dataset by utilizing convolutional layers that identify patterns and structures within the input data. While the CNN excels at capturing spatial relationships, it has limitations in understanding temporal dependencies due to its static processing of data. To address this, we integrate LSTM, which is specifically designed to handle sequential data and capture temporal dependencies, allowing it to retain information over time. By combining the strengths of both architectures, the hybrid CNN-LSTM model learns both spatial and temporal patterns, resulting in a more robust feature representation that enhances the model’s ability to detect intrusions in IoMT networks.

The CNN architecture consists of several layers, starting with an input layer that receives the selected network features, structured as (N, 1) for 1D CNNs when there are N features (32). The convolutional layer contains neurons with identical weights and biases, referred to as kernels or filters, which connect to a region of *n* × *n* neurons in the preceding layer. The output for the (*j*,*k*)th neuron is calculated as:


(3) $$yj,k=\overset{n-1}{\underset{l=0}{\sum }}\overset{n-1}{\underset{m=0}{\sum }}wl,m^{x}j+l,k+m+b$$

Following this is an activation layer, where a nonlinear activation function such as Rectified Linear Unit (ReLU) is applied to introduce nonlinearity into the model. ReLU, defined as $f\left(x\right)=\max\left(x,0\right)$ sets negative values in the feature maps to zero while preserving positive values, enhancing the model’s expressive power. The pooling layer then down-samples the output from the previous layer, typically using max-pooling to extract the maximum value from each non-overlapping sub-region. Finally, the flatten layer converts the high-dimensional feature maps into a 1D vector that is fed into the LSTM.

LSTM (33) is a type of recurrent neural network suitable for analyzing time series data. It can capture the temporal dependencies in data. LSTM can model long-term dependencies and correlations by consolidating memory units that can update a hidden state. The model is composed of four main components: a memory cell that is linked to itself, and three multiplicative units known as the input, output, and forget gates as in Figure 2. The LSTM, suitable for analyzing time series data, captures temporal dependencies through its architecture, which comprises four main components: a memory cell linked to itself and three multiplicative units known as the input, output, and forget gates (4). The LSTM processes the input sequence and captures these dependencies by receiving a flattened vector from the CNN. The equations governing the LSTM operations are as follows.


(4) $$i_{t}=\sigma \left(W_{i}.\;\left[h_{t-1},X_{t}\right]+b_{i}\right)$$

(5) $$f_{t}=\sigma \left(W_{f}.\;\left[h_{t-1},X_{t}\right]+b_{f}\right)$$

(6) $$C_{t}=\tanh\left(W_{c}.\;\left[h_{t-1},X_{t}\right]+b_{c}\right)$$

(7) $$O_{t}=\sigma \left(W_{o}.\;\left[h_{t-1},X_{t}\right]+b_{o}\right)$$

The final hidden state ht , which encapsulates the temporal features, is calculated as.


(8) $$ht=O_{t}\tanh\left(c_{t}\right)$$

The hybrid CNN-LSTM architecture aims to balance model complexity, learning rate, and regularization to effectively detect intrusions in the IoMT network environment. The tuning of these hyperparameters is crucial for optimizing the model’s performance and generalization capabilities.

### 3.3 Proposed reinforcement learning

**Figure 3:**
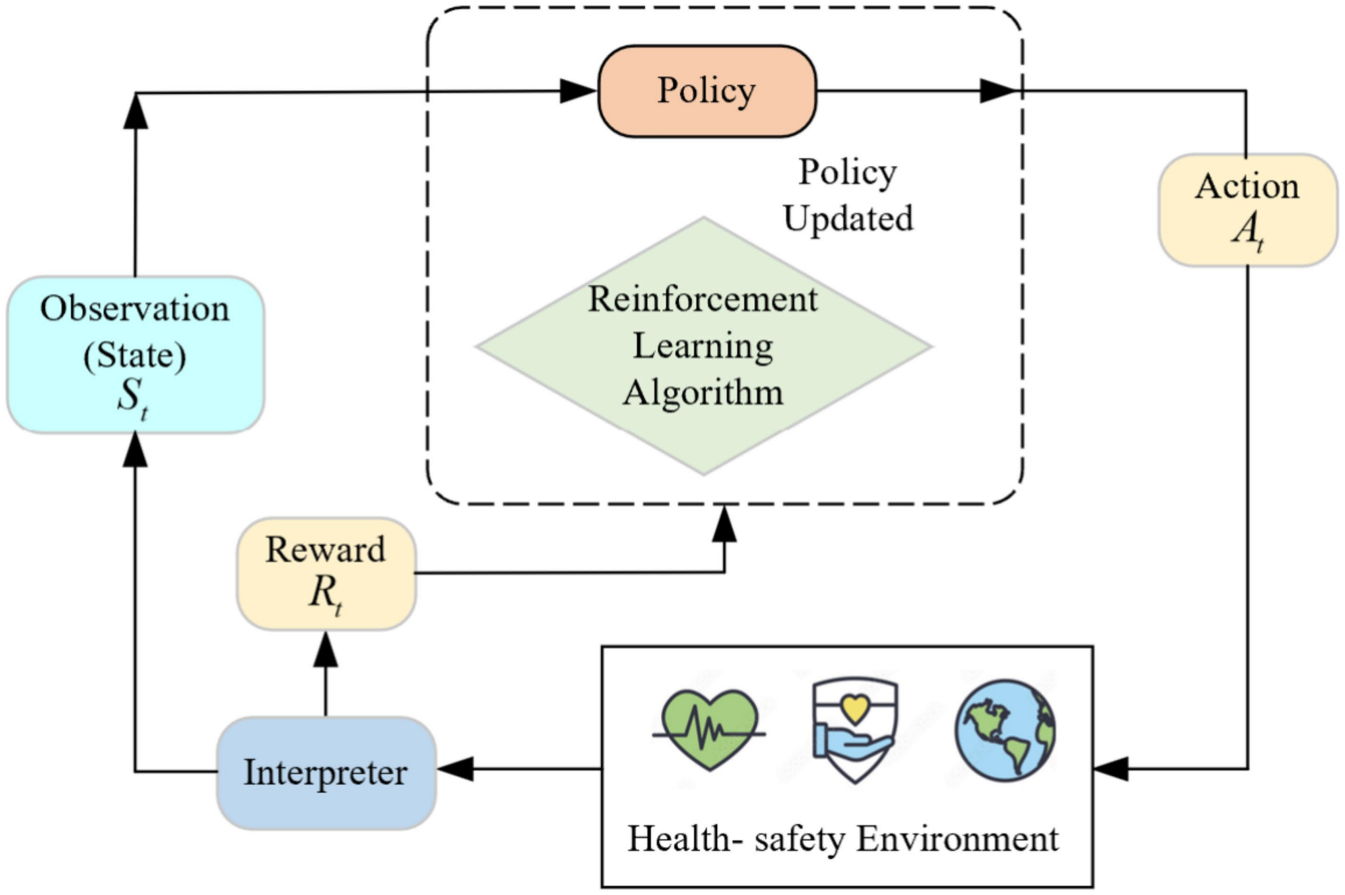
Proposed reinforcement learning architecture.

Reinforcement Learning (RL) (34) is a powerful machine learning approach designed to tackle decision-making and action-selection problems. In this framework, an agent interacts with an environment, learning to make optimal decisions through trial and error. The primary objective of reinforcement learning is to develop a policy that maximizes cumulative rewards over time. In reinforcement learning, the agent observes the state of the environment, performs actions, and receives feedback in the form of rewards. The agent’s actions are determined based on the current state, facilitating a dynamic interaction that leads to the next state and associated reward. This learning process can be formally represented as a Markov Decision Process (MDP) (35). A schematic diagram of the reinforcement learning process is illustrated in Figure 3.

The fundamental components of reinforcement learning can be outlined as follows:

1. *Reward Signal*: The agent receives immediate feedback from the environment based on its actions. Rewards can be positive, negative, or neutral, providing an evaluation of the action’s effectiveness.
2. *Policy*: The policy defines the strategy by which the agent selects actions based on the current state. It can be deterministic (assigning one specific action per state) or stochastic (using a probability distribution to select actions).
3. *Value Function*: The value function estimates the expected long-term cumulative reward for an agent following a specific policy. It aids in assessing the quality of different states and informs policy updates.
4. *Model*: A model represents the agent’s internal understanding of the environment, predicting state transitions and rewards. Methods that utilize a model are termed model-based, while those that operate without one are considered model-free.

The ultimate aim of reinforcement learning is to identify the optimal policy that maximizes cumulative rewards. The learning process typically involves repeated iterations where the agent interacts with the environment according to its current policy, gathers experience, and updates its value functions and policies based on this data. Common algorithms in reinforcement learning include DQN and PPO, among others. A distinctive feature of reinforcement learning is its ability to improve through exploration and interaction with the environment, fostering autonomous decision-making and intelligent behavior.

In the proposed work, we utilize RL for intrusion detection, where we define the key components of states, actions, and rewards as follows.

- States: The states in our RL model are represented by feature vectors derived from the outputs of the CNN and LSTM models. These feature vectors capture both spatial and temporal information about the network traffic, providing a comprehensive representation of the current state of the system.
- Actions: The actions correspond to the decisions made by the intrusion detection system (IDS), such as classifying the network traffic as either normal or malicious. The RL agent learns which actions lead to the most accurate detections of various attack types.
- Rewards: The rewards are assigned based on the accuracy of the model’s classification. If the model correctly classifies the traffic (either as normal or malicious), a positive reward is given. Conversely, incorrect classifications (false positives or false negatives) result in negative rewards. The objective of the RL agent is to maximize cumulative rewards over time by learning the optimal policy for intrusion detection.

This RL framework enables the system to adapt dynamically to new attack patterns by continually improving its decision-making process based on feedback from previous actions. This continual learning process ensures that the IDS is capable of handling novel, evolving attack vectors.

#### 3.3.1 Proposed DQN model

The DQN (36) is a specific reinforcement learning algorithm that focuses on learning decision-making strategies by approximating Q-values. These Q-values represent the expected future rewards associated with taking a specific action in a given state. The DQN algorithm iteratively refines an optimal Q-function to maximize cumulative rewards. In the context of an intrusion detection system, the state is represented by a feature vector derived from network traffic data, specifically produced by a Long LSTM network that captures both spatial and temporal dependencies.

We use the *Q*-function, which represents the expected cumulative reward when starting from the state s , taking action a , and subsequently following the policy *π*. This is defined as.


(9) $$Q^{\pi }\left(s,a\right)=E\left[R_{t}|s_{t}=s,a_{t}=a,\pi \right]$$

Here, $R_{t}$ is the cumulative reward from the time step t onward.

The state-value function $V^{\pi }\left(s\right)$ is the expectation of the *Q*-value over all possible actions a that could be taken from the state s according to the policy π . It is defined as:


(10) $$V^{\pi }\left(s\right)=E_{a\sim \pi \left(s\right)}\left[Q^{\pi }\left(s,a\right)\right]$$

The optimal *Q*-function *Q*∗(*s*, *a*) can be expressed in the following manner.


(11) $$Q^{*}\left(s,a\right)=\max_{\pi }Q^{\pi }\left(s,a\right)$$

For optimal *Q*-function, the optimal state-value function $V^{*}\left(s\right)$ is.


(12) $$V^{*}\left(s\right)=\max_{a}Q^{*}\left(s,a\right)$$

We define the state-dependent action advantage function to measure how much better or worse taking a specific action a in state s is compared to the average expected outcome of being in that state. It is defined as.


(13) $$A^{\pi }\left(s,a\right)=Q^{\pi }\left(s,a\right)-V^{\pi }\left(s\right)$$

By subtracting $V^{\pi }\left(s\right)$ from $Q^{\pi }\left(s,a\right)$ , we get $A^{\pi }\left(s,a\right)$ , which tells us how much better action a is compared to the average action according to the policy π . We define the loss function to minimize the difference between the predicted *Q*-values and the target *Q*-values. We accumulate experience from every iteration, store it in a replay buffer, and then use these stored experiences to update the *Q*-network. The loss function is given as.


(14) $$L_{i}\left(\theta_{i}\right)=E_{\left(s,a,r,s^{\prime }\right)\sim U\left(D\right)}\left[{\left(\begin{array}{l} r+\gamma_{a^{\prime }}^{\text{max}}Q\left(s^{\prime },a^{\prime };\theta_{i}^{-}\right)-\\ Q\left(s,a;\theta_{i}\right) \end{array}\right)}^{2}\right]$$

This loss function represents the sum of squared errors between the target network’s predicted future reward and the *Q*-network’s predictions. The *Q*-network continues to improve as it updates its target network with new information over time. To address challenges associated with limited sample sizes and correlations between training samples, DQNs employ experience replay, enhancing data efficiency by reusing empirical samples across multiple updates. This method reduces variance and promotes uniform sampling from the replay buffer, thereby minimizing correlations among samples used during updates. The DQN algorithm steps are detailed in Algorithm 1.

#### 3.3.2 Proposed PPO model

PPO (37) enhances a stochastic policy through iterative updates, employing a clipped surrogate objective to balance exploration and exploitation while ensuring stable learning. One of the key benefits of PPO is its ability to maximize exploration without significantly increasing the algorithm’s computational complexity. The policy is updated using the policy gradient theorem to enhance expected rewards.

In PPO, the agent comprises two main components: the actor and the critic. The actor’s role is to determine the optimal policy to maximize rewards based on the environment. The actions generated by the actor can be represented as.


(15) $$a_{k}\sim \pi_{\theta }\left(s_{k}\right)$$

The critic module evaluates the agent’s actions by estimating the value function of the system’s state $V_{\mu }\left(s_{k}\right)$ , which predicts the expected cumulative reward


(16) $$V_{\mu }\left(s_{k}\right)=E\left[R|s_{k}\right]$$

PPO utilizes a modified objective function to iteratively improve the target function $L^{\mathrm{clip}}$ by clipping updates to keep the new policy close to the old one. This prevents large policy changes that could destabilize training. The update rule for the policy is given by.


(17) $$\theta_{k+1}=\arg\max_{\theta }E_{s,a\sim \pi \theta_{k}}\left[L^{\mathrm{clip}}\left(s,a,\theta_{k},\theta \right)\right]$$

Here, $L^{\mathrm{clip}}\left(s,a,\theta_{k},\theta \right)$ represents the surrogate advantage function, which assesses the new policy’s performance relative to the old policy:


(18) $$L^{\mathrm{clip}}\left(s,a,\theta_{k},\theta \right)=\min\left(\begin{array}{l} \frac{\pi_{\theta }\left(a|s\right)}{\pi_{\theta_{k}}\left(a|s\right)}A^{\pi_{\theta_{k}}}\left(s,a\right),\\ \mathrm{clip}\left(\frac{\pi_{\theta }\left(a|s\right)}{\pi_{\theta_{k}}\left(a|s\right)},1-\in ,1+\in \right)\\ A^{\pi_{\theta_{k}}}\left(s,a\right) \end{array}\right)$$

Where ∈ is a (small) hyperparameter, which roughly says how far away the new policy is allowed to go from the old. The former policy is denoted by $\pi_{\theta_{k}}$ in the equation above, while the current policy is represented by $\pi_{\theta }\text{.}$ As a result, $\pi_{\theta }\left(a|s\right)$ represents the likelihood that states will behave according to the present policy. Moreover, $A^{\pi_{\theta_{k}}}\left(s,a\right)$ denotes the Advantage that is, the projected gain in the reward that is noticed when a state employs policy $\pi_{\theta_{k}}$ to do action a . The objective of the advantage function is to provide a relative measure of the goodness of an action, rather than an absolute value.

In this equation, ∈ is a hyperparameter that determines the allowable deviation of the new policy from the old one. The advantage function $A^{\pi_{\theta_{k}}}\left(s,a\right)$ serves to indicate the relative benefit of an action in a given state.

The objective function (L) and the ratio of the two distributions (r) are depicted in figures illustrating the PPO optimization process. When the advantage is positive, the updates encourage actions that yield higher rewards, while negative advantages drive the agent away from suboptimal actions. The implementation details of the PPO algorithm are outlined in Algorithm 2.


(19) $$A^{\pi }\left(s,a\right)=Q^{\pi }\left(s,a\right)-V^{\pi }\left(s\right)$$

There is another simplified version of this objective.


(20) $$L\left(s,a,\theta_{k},\theta \right)=\min\left(\frac{\pi_{\theta }\left(a|s\right)}{\pi_{\theta_{k}}\left(a|s\right)}A^{\pi_{\theta_{k}}}\left(s,a\right),\delta \left(\in ,A^{\pi_{\theta_{k}}}\left(s,a\right)\right)\right)\text{,}$$

Where $\delta \left(\in ,A\right)$ is defined as.


(21) $$\delta \left(\in ,A\right)=\{\begin{array}{c} \left(1+\in \right)A\;A\geq 0\\ \left(1-\in \right)A\;A<0\; \end{array}$$

In the above equation, a positive benefit suggests that the agent took a wise course of action. Therefore, we would want the update to further inspire the choice of that course of action. But we do not want the update to be too big.

**Figure 4:**
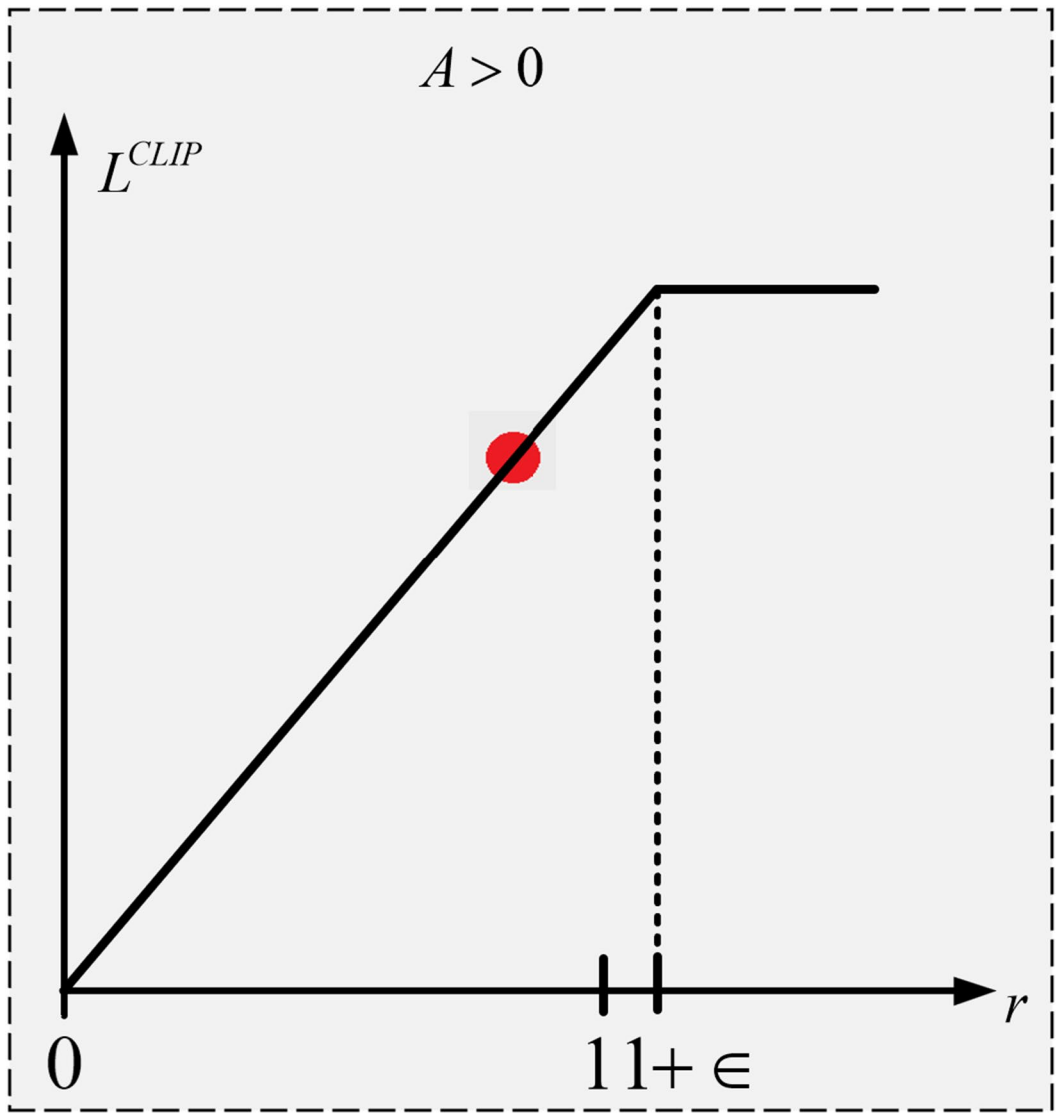
PPO objective function with +ve advantage.

**Figure 5:**
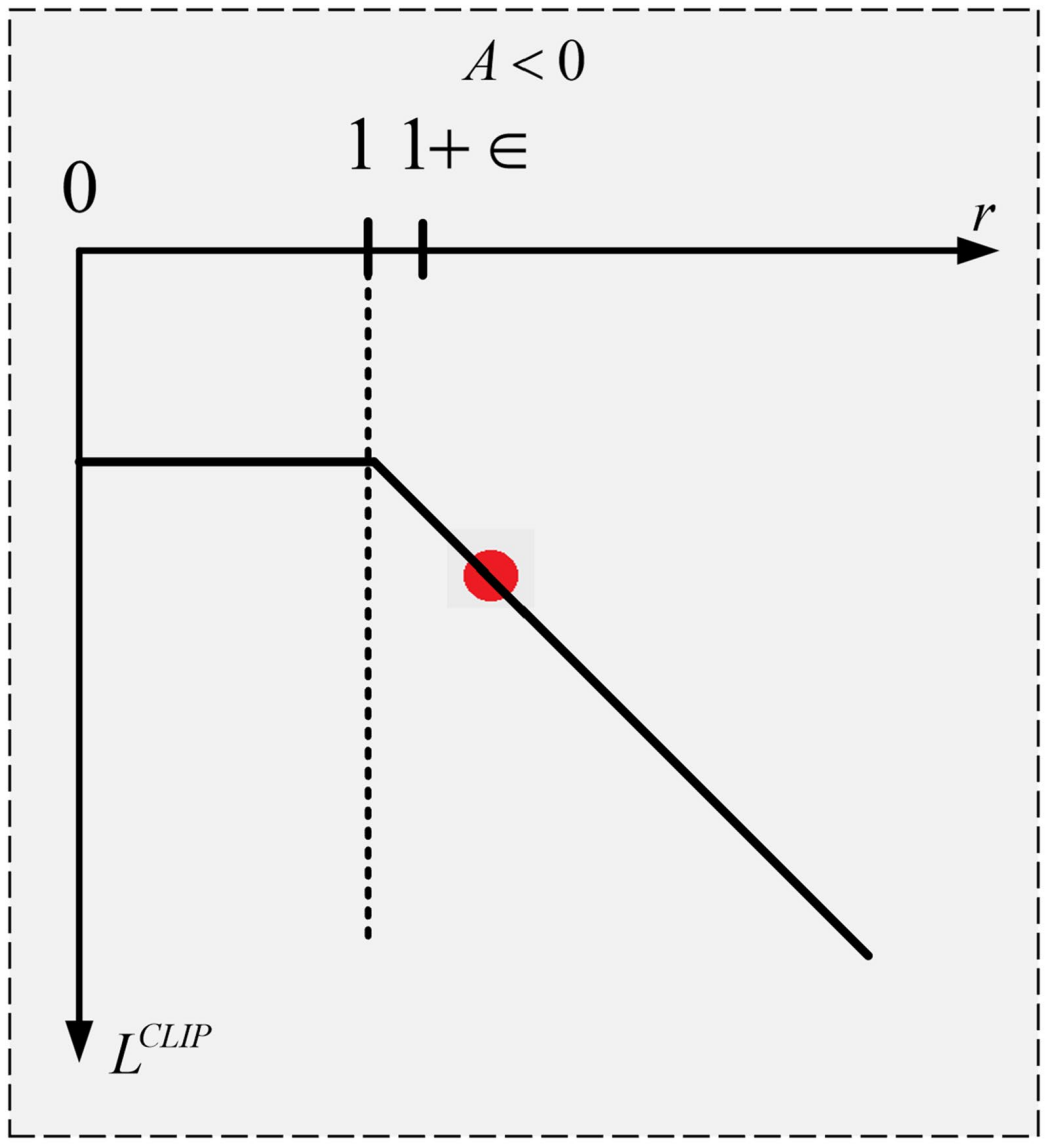
PPO objective function with −ve advantage.

The objective function $\left(L\right)$ and the ratio between the two distributions (r ) are represented by the x and y axes, respectively, in Figure 4. The goal function’s value will be rA as long as r is less than or equal to 1+∈ . Thus, in this instance, the ratio of 1+∈ corresponds to the maximum of the objective function. Since we are choosing the minimum, 1-*ρ* does not matter, therefore even if rA is smaller than $\left(1+\in \right)rA\text{,}$ it will not be clipped. This is because we do not care if the update is too little. Whereas a negative advantage suggests that the agent acted improperly. Therefore, for the specified state, we want the update to make the agent less likely to do that action. Stated otherwise, the updates will be in the opposite direction as the goal function now has a negative value as shown in Figure 5. The objective function will have a value of $\left(1+\in \right)A$ as long as r is larger than or equal to $\left(1+\in \right)\text{.}$ Thus, in this instance, the ratio of $\left(1+\in \right)$ corresponds to the maximum of the objective function. The goal function’s value becomes increasingly negative as r rises above $\left(1+\in \right)\text{.}$ This indicates that a reduced number of policy changes will result from the update’s attempt to prevent it.

The implementation of the algorithm is shown in Algorithm 2.

##### ALGORITHM 1 Proposed DQN


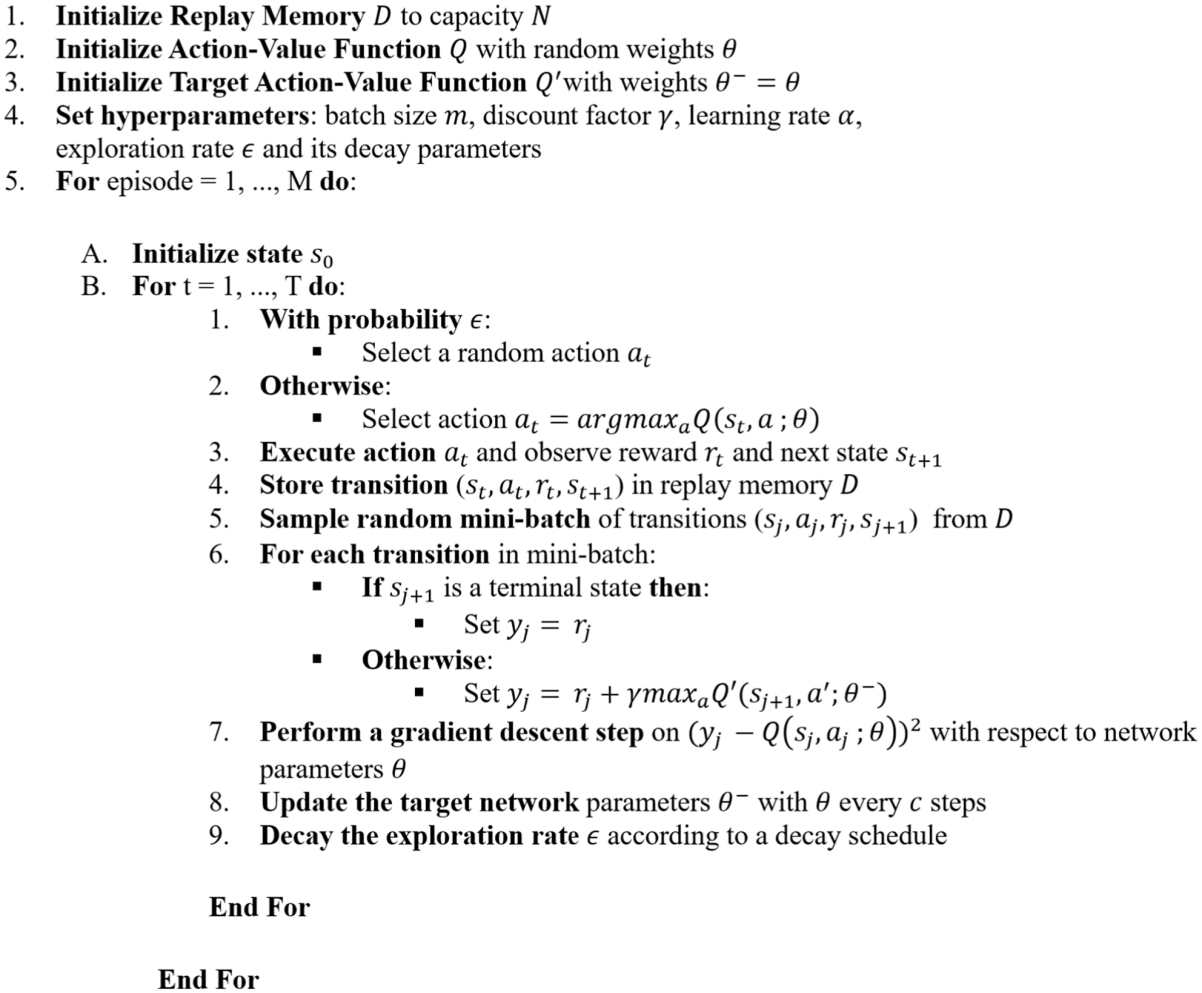


##### ALGORITHM 2 Proposed PPO


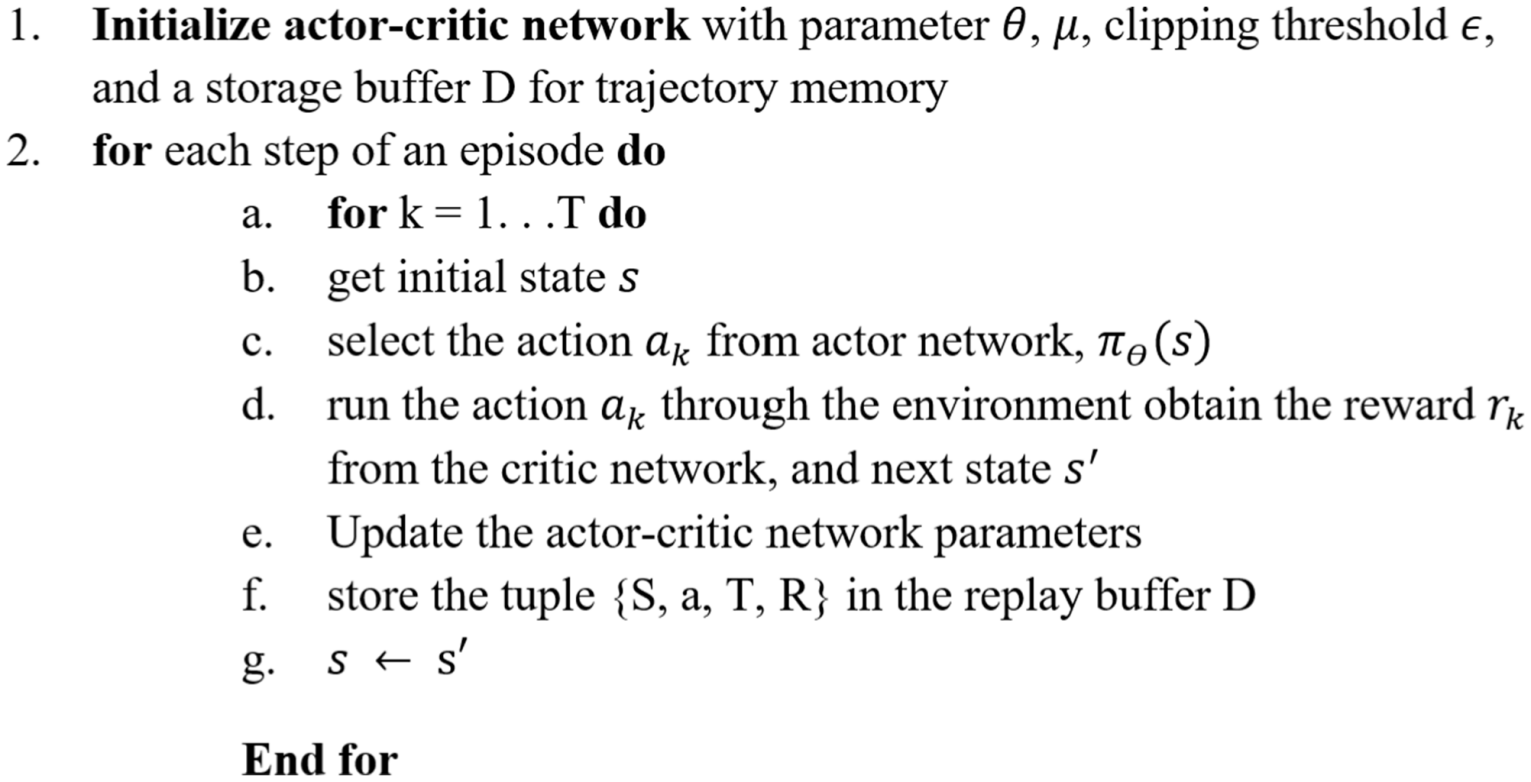


## 4 Experimental setup

To address concerns about overfitting and data leakage, we implemented 5-fold cross-validation to ensure consistent model performance across different subsets of the data. In this approach, the dataset was split into five folds, where each fold served as both a training and testing set. This ensured that no single partition influenced the model’s performance evaluation. Our results consistently showed high accuracy across all folds, demonstrating that the model is generalizable and not overfitting to specific data subsets. To further prevent data leakage, we ensured strict separation of data preprocessing steps for training and testing. Feature selection and normalization were performed independently on the training and test sets to avoid any overlap that could artificially inflate performance metrics. The model’s consistent performance across validation folds validates the reliability of our results, confirming they were not impacted by data leakage. For the experimental setup, the CICIoMT2024 dataset was divided into three subsets: 70% for training, 10% for validation, and 20% for testing. The training set allowed the model to learn patterns from the data, while the validation set was used for hyperparameter tuning and performance monitoring to prevent overfitting. The test set, which remained unseen during training, was used for final evaluation to assess real-world performance. Additionally, to improve the model’s robustness and generalization, 5-fold cross-validation was employed. In this method, the dataset was divided into five equal subsets, and the model was trained on four folds while being tested on the remaining fold. This process was repeated for all five combinations, and the final performance metrics were averaged to reduce variance and ensure that the model’s performance was not biased by any specific data split.

### 4.1 Hyper optimization

In this study, hyperparameter tuning for the proposed HCLR-IDS was performed using a grid search approach. This method exhaustively searches through a predefined set of hyperparameters to identify the optimal configuration for the model. We tested various combinations of hyperparameters, including learning rate, batch size, dropout rate, and the number of CNN filters. For the reinforcement learning models, we also optimized the discount factor, exploration rate decay, and the number of training epochs. The goal was to strike a balance between model performance and computational efficiency. The rationale behind the selection of these hyperparameters is detailed in the Experimental Setup section.

Grid search is particularly effective for small search spaces, as it systematically evaluates all possible combinations of hyperparameters, ensuring that the best-performing set is identified. For the proposed HCLR-IDS, which integrates CNN and LSTM networks with Deep Q-Networks (DQN) and Proximal Policy Optimization (PPO), the following hyperparameters were tested:

- *CNN*: Learning rate (0.001, 0.0001), number of filters (32, 64, 128), kernel size (3×3, 5×5), dropout rate (0.2, 0.3), batch size (32), and epochs (100).
- *LSTM*: Learning rate (0.001, 0.0001), number of units (64, 128, 256), dropout rate (0.2, 0.3), batch size (32), and epochs (100).
- *DQN*: Learning rate (0.001, 0.0001), discount factor (0.99, 0.95), exploration rate decay (0.995, 0.999), batch size (32), and epochs (100).
- *PPO*: Learning rate (0.0003, 0.0001), clip range (0.1, 0.2), number of epochs for policy update (3, 5), batch size (32), and epochs (100).

These hyperparameters were selected to optimize the model’s performance while ensuring computational efficiency, thereby enhancing the robustness of intrusion detection in IoMT healthcare networks.

### 4.2 Software and hardware preliminaries

Our studies were carried out on a computer powered by an AMD Ryzen 53,600 CPU running at 4.2GHz, 128GB of RAM, and an Nvidia RTX 3060 Ti graphics card. To make use of the GPU’s processing capabilities, we used Nvidia CUDA version 11.3. The operating system was Windows 10 Pro, and the software stack included Python 3.10.0, TensorFlow 2.9.1 for deep learning, Scikit-learn 1.1.3 for machine learning tasks, Matplotlib 3.5.3 for data visualization. This combination of hardware and software provides the resources required to properly train, test, and analyze the deep learning models utilized in our study.

### 4.3 Model evaluation

The evaluation of classification models is essential for understanding their performance in real-world applications, particularly in complex domains like intrusion detection in Internet of Medical Things (IoMT) networks. In this study, we focus on several key metrics: accuracy, precision, recall (sensitivity), and F1 score. These metrics are derived from counts of True Positives (TP), True Negatives (TN), False Positives (FP), and False Negatives (FN), which are obtained from the confusion matrix. The confusion matrix provides a clear overview of how well the model is performing against each class. Mathematically, the evaluation metrics are defined as follows.


(22) $$\mathrm{Accuracy}=\frac{TP+TN}{TP+TN+FP+FN}$$

(23) $$\mathrm{Precision}=\frac{TP}{TP+FP}$$

(24) $$\mathrm{Recall}=\frac{TP}{TP+FN}$$

(25) $$F1\mathrm{Score}=2\times \frac{\left(\mathrm{Precision}\times \mathrm{Recall}\right)}{\mathrm{Precision}+\mathrm{Recall}}$$

The ROC curve visualizes the relationship between the true positive rate (TPR) and the false positive rate (FPR) across different threshold settings. The Area Under the Curve (AUC) quantifies the model’s ability to differentiate between classes. While there is not a single equation for the AUC, it is derived from the integral of the ROC curve, usually computed using numerical methods. Additionally, a confusion matrix is a valuable tool for assessing the performance of a classification model. It summarizes the counts of true positives (TP), true negatives (TN), false positives (FP), and false negatives (FN), providing a clear overview of the model’s predictive accuracy.

## 5 Results and discussion

**Figure 6:**
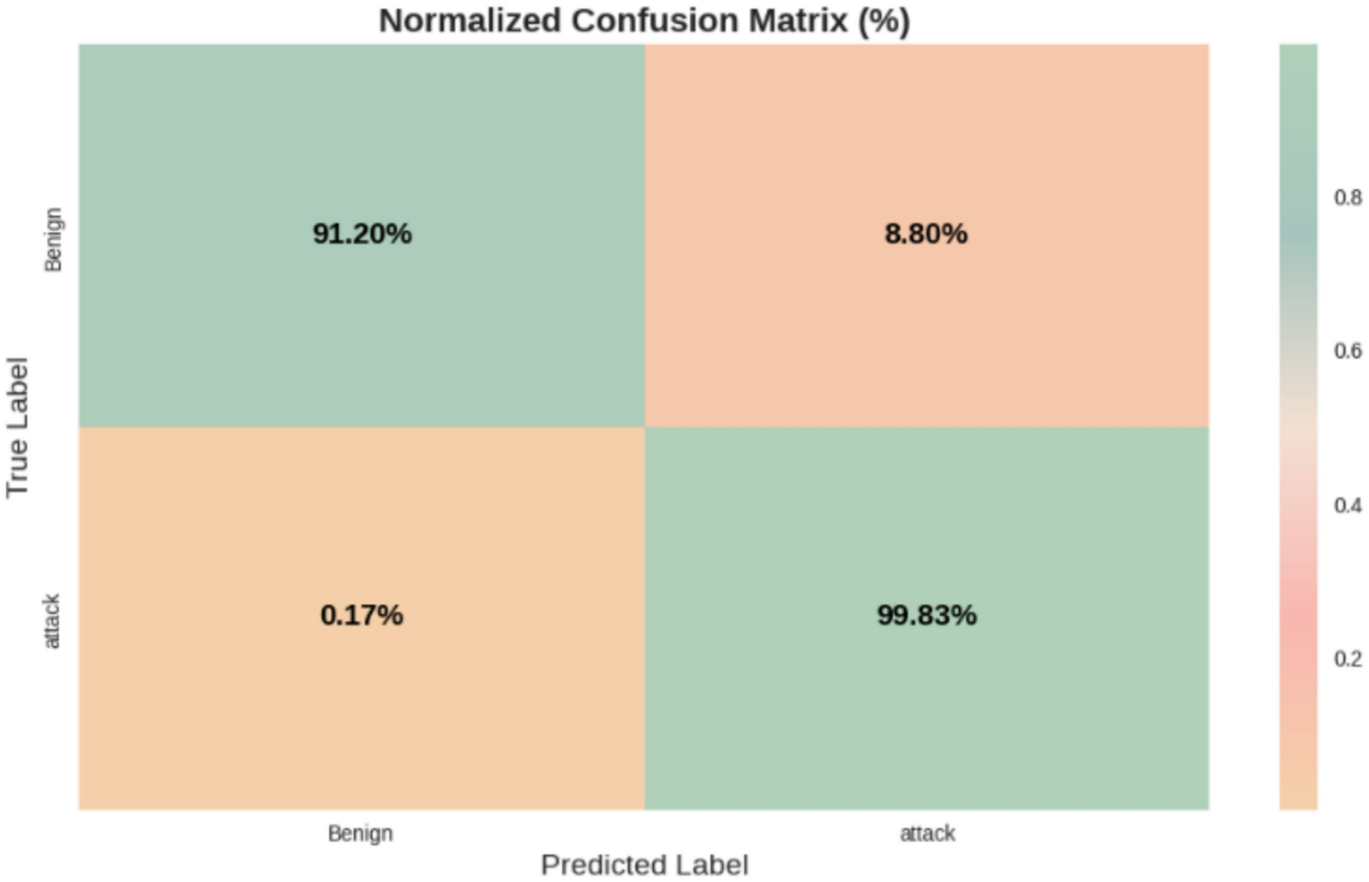
Confusion matrix of the proposed HCLR-IDS for binary classification.

**Figure 7:**
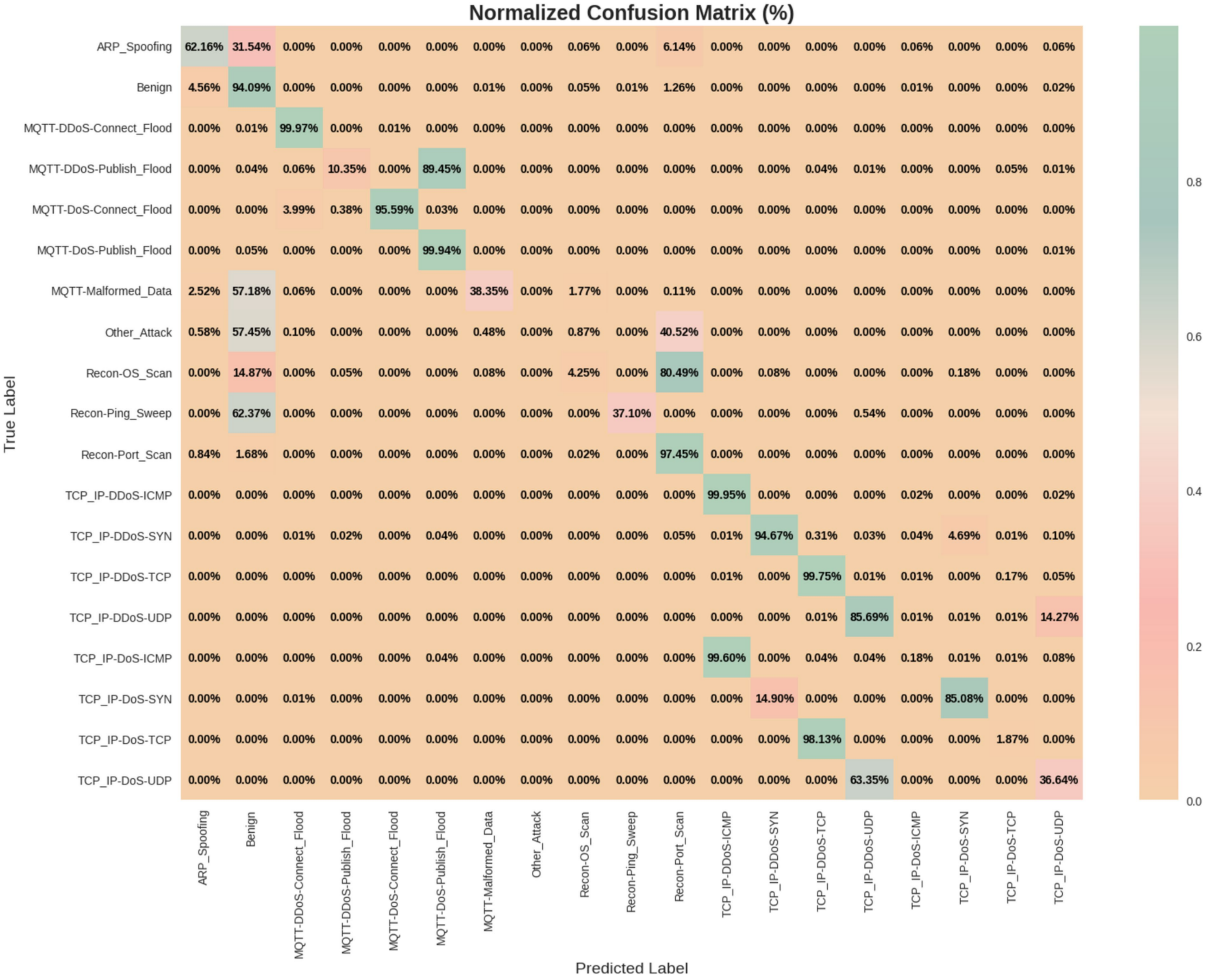
Confusion matrix of the proposed HCLR-IDS for multiclassification.

The experimental results of our study are presented in Table 3 and illustrated in Figure 6, where we compare various models using performance metrics such as accuracy, precision, recall, F1 score, and Area Under the Curve (AUC). Accuracy indicates the proportion of correctly classified samples, while recall assesses the model’s ability to identify positive samples. The F1 score provides a balance between precision and recall, while Figure 7 represents the AUC-ROC, highlighting the model’s classification capability. Together, these metrics offer a comprehensive evaluation of the models’ performance in intrusion detection.

**Table 3:** Proposed HCLR-IDS with and without features selection.

Model	Accuracy	Precision	Recall	F1-score
Before feature selection	0.6973	0.5680	0.7666	0.6525
After features selection	0.9958	0.9953	0.9983	0.9957

The study utilized the CICIoMT2024 dataset, which comprises labeled network traffic data tailored for IoMT applications. To improve the quality of the data for intrusion detection, several preprocessing steps were undertaken, including handling missing values, feature scaling using min-max normalization, and encoding categorical features. Additionally, the Mutual Information Feature Selection (MIFS) technique was applied to reduce the feature space from 42 to the most relevant 34 features. Table 3 summarizes the performance metrics of the proposed HCLR-IDS both before and after feature selection. Initially, the model achieved an accuracy of 69.73%, with precision, recall, and F1-score values of 56.80, 76.66, and 65.25%, respectively. After applying feature selection, the model demonstrated a significant improvement, achieving an accuracy of 99.58%, along with precision, recall, and F1-score values of 99.53, 99.83, and 99.57%, respectively.

MIFS was critical for this enhancement. Traditional intrusion detection methods often struggle with high-dimensional data, as they may fail to identify the most relevant features, resulting in lower performance and increased computational costs. By utilizing MIFS, we were able to select only the most informative features that maximized the mutual information with the target variable (i.e., attack classes). This not only improved the accuracy of the model but also reduced the complexity and computational demands, making the system more efficient. The application of MIFS addressed the limitations of traditional methods by eliminating irrelevant or redundant features, which are often problematic in high-dimensional datasets. These traditional methods could either overlook critical patterns in the data or overfit to noise, leading to suboptimal detection performance. By applying MIFS, the model became more focused on the most pertinent features, leading to improved detection effectiveness and computational efficiency. This demonstrates how feature selection is essential for improving the performance of intrusion detection systems (IDS) in complex IoMT environments, where data quality and system efficiency are paramount.

Tables 4, 5 present the performance metrics of the proposed HCLR-IDS for binary and multiclass classification tasks, respectively. In binary classification (Table 4), HCLR-IDS achieves 0.9958 accuracy, with perfect precision, recall, and F1-score for the attack class, demonstrating strong detection capabilities. For multiclass classification (Table 5), the model excels across various attack types, achieving an overall accuracy of 0.7773, with high precision and recall for many attack classes, showcasing its robustness in identifying diverse cyber threats. Figures 6, 7 display the confusion matrix visualizations for binary and multiclass classifications, respectively, providing a detailed view of the model’s performance for each class.

**Table 4:** Binary classification of the proposed HCLR-IDS.

Class	Label	Matric	HCLR-IDS
Benign	0	Precision	0.9312
		Recall	0.9127
		F1-score	0.9247
Attack	1	Precision	1
		Recall	1
		F1-score	1
Accuracy	0.9958		
Macro precision	0.9881		
Macro recall	0.9821		
Macro F1-score	0.9857		
Weighted precision	0.9953		
Weighted recall	0.9983		
Weighted F1-score	0.9957		

**Table 5:** Multiclassification of the proposed HCLR-IDS.

Class	Accuracy	Precision	Recall	F1-score
ARP_Spoofing	0.62	0.36	0.62	0.45
Benign	0.94	0.92	0.94	0.93
MQTT-DDoS-Connect_Flood	1.00	1.00	1.00	1.00
MQTT-DDoS-Publish_Flood	0.10	0.94	0.10	0.19
MQTT-DoS-Connect_Flood	0.96	1.00	0.96	0.98
MQTT-DoS-Publish_Flood	1.00	0.53	1.00	0.69
MQTT-Malformed_Data	0.38	0.98	0.38	0.55
Other_Attack	0.00	0.00	0.00	0.00
Recon-OS_Scan	0.04	0.71	0.04	0.08
Recon-Ping_Sweep	0.37	0.91	0.37	0.53
Recon-Port_Scan	0.97	0.84	0.97	0.90
TCP_IP-DDoS-ICMP	1.00	0.78	1.00	0.88
TCP_IP-DDoS-SYN	0.95	0.92	0.95	0.93
TCP_IP-DDoS-TCP	1.00	0.69	1.00	0.82
TCP_IP-DDoS-UDP	0.86	0.78	0.86	0.82
TCP_IP-DoS-ICMP	0.00	0.49	0.00	0.00
TCP_IP-DoS-SYN	0.85	0.91	0.85	0.88
TCP_IP-DoS-TCP	0.02	0.80	0.02	0.04
TCP_IP-DoS-UDP	0.37	0.49	0.37	0.42
Accuracy	0.7773			
Macro precision	0.7386			
Macro recall	0.6016			
Macro F1-score	0.5829			
Weighted precision	0.7602			
Weighted recall	0.7773			
Weighted F1-score	0.7247			

### 5.1 Ablation study on CICIoMT2024 dataset

**Figure 8:**
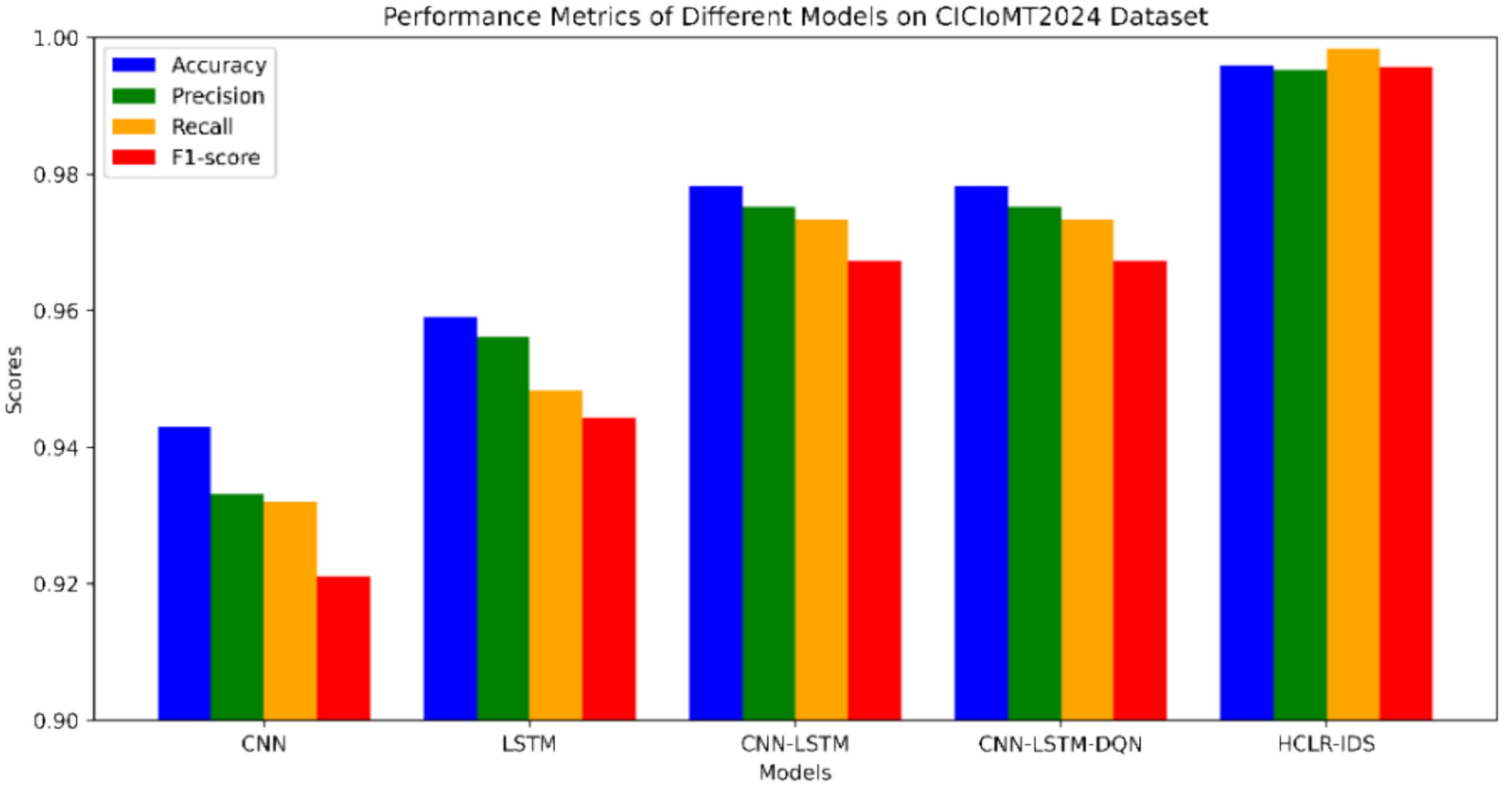
Performance of models in CICIoMT2024 dataset.

Table 6 and Figure 8 presents the results of an ablation study conducted on the CICIoMT2024 dataset, evaluating the performance of various models, CNN-only, LSTM-only, Hybrid CNN-LSTM (without RL), Hybrid CNN-LSTM with DQN, and Hybrid CNN-LSTM with PPO. The performance metrics—accuracy, precision, recall, and F1-score demonstrate significant improvements as we progressively combine different model components.

**Table 6:** Ablation study on CICIoMT2024 dataset.

CNN	LSTM	CNN-LSTM	DQN	PPO	HCLR-IDS	Accuracy	Precision	Recall	F1-score
✓	☓	☓	☓	☓	☓	0.9091	0.9535	0.8758	0.9130
☓	✓	☓	☓	☓	☓	0.9430	0.9331	0.9321	0.9211
✓	✓	✓	☓	☓	☓	0.9591	0.9561	0.9482	0.9443
✓	✓	✓	✓	☓	☓	0.9783	0.9752	0.9733	0.9672
✓	✓	✓	✓	✓	✓	0.9958	0.9953	0.9983	0.9957

The CNN-only model achieved an accuracy of 90.91%, but it struggled with false negatives, leading to a lower recall of 87.58%, highlighting its limitations in capturing temporal dependencies. In contrast, the LSTM-only model performed better, with 94.30% accuracy and 93.21% recall, as it excelled in recognizing sequential patterns. However, LSTM models lack the ability to capture spatial features, which are essential for intrusion detection in complex network traffic scenarios. The Hybrid CNN-LSTM model, which combines both spatial and temporal features, improved accuracy to 95.91% but lacked the adaptability required for real-time decision-making, which is critical for dynamic IoMT environments.

Incorporating Deep Q-Network (DQN) into the Hybrid CNN-LSTM model improved performance further, reaching 97.83% accuracy. DQN enabled adaptive learning from past experiences, optimizing decisions based on evolving network conditions. However, DQN models can be computationally intensive and require extensive training data, which can limit their real-time applicability. Finally, the Hybrid CNN-LSTM with PPO (HCLR-IDS) model achieved the best performance, with an impressive 99.58% accuracy, 99.53% precision, 99.83% recall, and 99.57% F1-score. PPO improved decision-making stability, ensuring the model adapts efficiently to new data while preventing large policy updates that could destabilize learning. By combining CNN, LSTM, DQN, and PPO, HCLR-IDS captures both spatial and temporal features, provides adaptive learning, and maintains stable performance, making it the most effective model for intrusion detection in IoMT networks.

**Figure 9:**
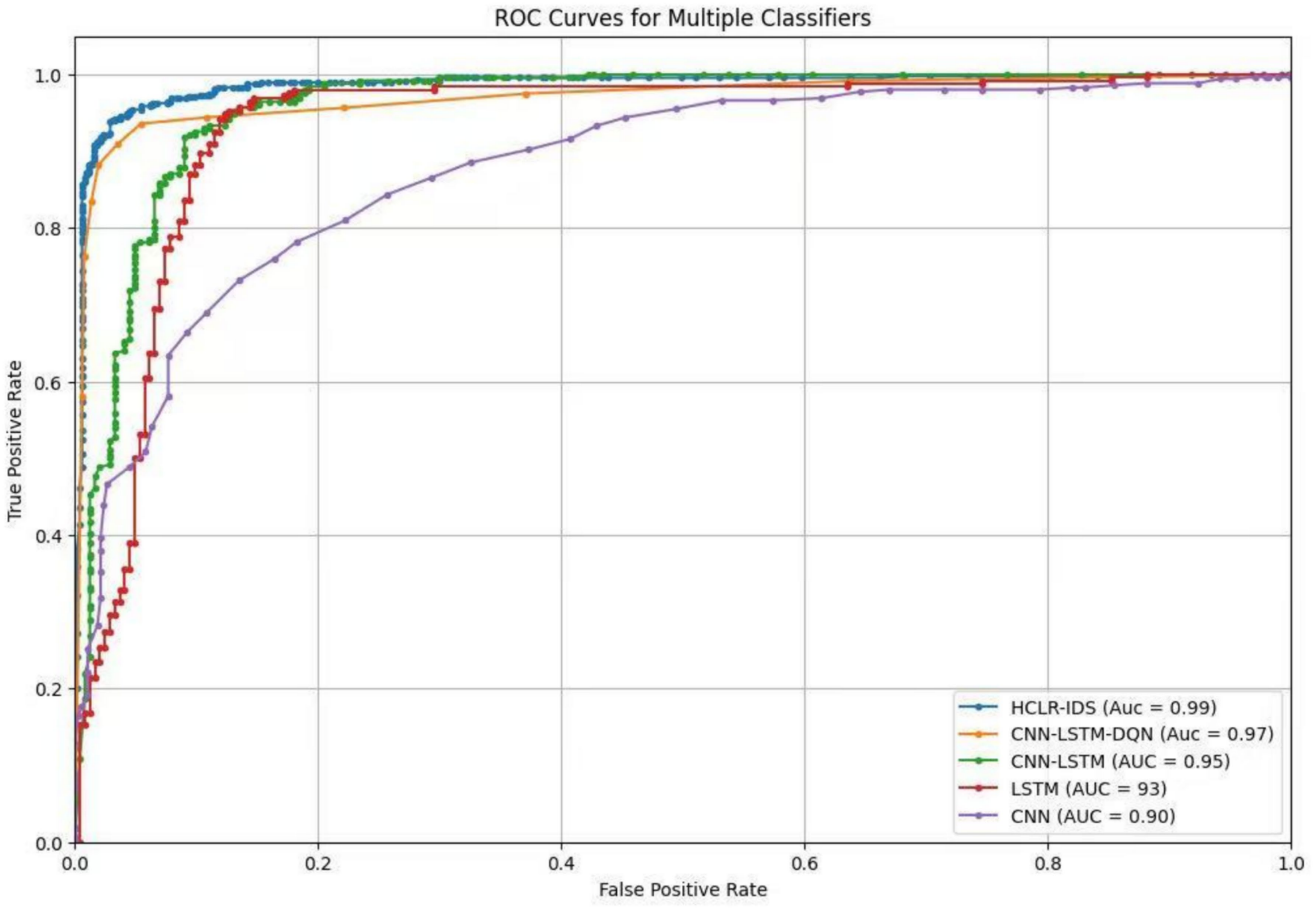
ROC curves.

The ROC curve analysis in Figure 9 showcases the performance of multiple classifier models employed in the proposed Hybrid Convolutional and Reinforcement Learning Intrusion Detection System (HCLR-IDS) for IoMT networks. The HCLR-IDS model demonstrates the highest Area Under the Curve (AUC) value of 0.99, highlighting its superior ability to accurately distinguish between normal network traffic and various attack types. This outstanding performance underscores the effectiveness of the hybrid approach, which combines the spatial and temporal analysis capabilities of CNN and LSTM networks.

The CNN-LSTM-DQN model, which further integrates a Deep Q-Network (DQN) component, also exhibits strong classification performance with an AUC of 0.97. The integration of these diverse architectures, including CNN, LSTM, and DQN, enables the model to capture both spatial and temporal patterns in the network traffic data, as well as leverage reinforcement learning techniques for enhanced decision-making. The standalone CNN-LSTM model, without the DQN component, achieves an AUC of 0.95, underscoring the benefits of combining convolutional and recurrent neural networks to address the complex characteristics of IoMT network traffic.

The LSTM model, with an AUC of 0.93, demonstrates the importance of effectively modeling the temporal dependencies in the data, which is crucial for identifying intrusions that may manifest over time. In contrast, the CNN model, with the lowest AUC of 0.90, highlights the limitations of relying solely on spatial feature extraction without considering the temporal aspects of network traffic patterns. The comparative analysis of these ROC curves and AUC values provides valuable insights into the strengths and weaknesses of the various approaches, guiding the selection of the most suitable intrusion detection solution for IoMT.

### 5.2 Comparison of our proposed HCLR-IDS with previous approaches

Table 7 presents a comparative analysis of the performance of our proposed HCLR-IDS approach against several well-established techniques documented in the literature, evaluated on the CICIoMT2024 dataset. The results show that HCLR-IDS significantly outperforms all previous models, achieving 99.58% accuracy, 99.53% precision, 99.83% recall, and 99.57% F1-score. These metrics clearly highlight the superior effectiveness of our hybrid approach in detecting and classifying various cyberattacks, such as Distributed Denial of Service (DDoS) and spoofing.

**Table 7:** Different methods on CICIoMT2024 dataset.

Article	Techniques	Accuracy	Precision	Recall	F1-score
(30)	LR	0.995	0.94	0.952	0.946
(38)	Transformer	0.79	0.80	0.79	0.78
(39)	MultiD-CNN	0.9672	0.9556	0.9665	0.9542
(40)	CNN	0.9950	0.9950	0.9950	0.9950
(41)	LSTM	0.989	0.99	0.988	0.989
(42)	GBM	0.9780	0.979	0.978	0.978
(43)	Ensemble Learning (RF+ SVM)	0.982	0.982	0.981	0.981
Our	HCLR-IDS	0.9958	0.9953	0.9983	0.9957

In comparison to traditional models such as Logistic Regression (LR) from Ref. (30), which reports 99.5% accuracy, 94% precision, 95.2% recall, and 94.6% F1-score, our model outperforms it across all metrics. While the LR model performs well, its performance is limited by its inherent simplicity and its reliance on linear relationships between features, which are insufficient for capturing the complex spatial and temporal patterns present in IoMT network traffic. In contrast, HCLR-IDS integrates CNN, LSTM, DQN, and PPO models, which allows it to extract both spatial and temporal features and adapt to changing patterns dynamically, enhancing its overall detection capability.

The Transformer and LSTM models from Ref. (38) show much lower results, with Transformer achieving only 79% accuracy and LSTM achieving 68% accuracy. These models suffer from limitations in capturing both spatial and temporal dependencies effectively. While LSTM is strong in sequential data processing, it struggles to account for spatial features essential in analyzing complex network traffic patterns. On the other hand, Transformers have difficulty processing the sequential nature of network traffic without the sequential and spatial interplay needed for high-performance intrusion detection in IoMT systems. Moreover, the MultiD-CNN model from Ref. (39)achieves a commendable accuracy of 96.72%, but HCLR-IDS still outperforms it. The MultiD-CNN model focuses on CNNs for spatial feature extraction but lacks the adaptive learning capabilities that DQN and PPO offer in HCLR-IDS. These reinforcement learning models allow HCLR-IDS to continuously adapt to new and evolving attack patterns, making it more resilient to the dynamic and real-time nature of IoMT networks.

Additionally, HCLR-IDS significantly outperforms CNN-based models such as the one in Ref. (40), which achieves 99.5% accuracy, 99.5% precision, 99.5% recall, and 99.5% F1-score. While CNNs are effective for spatial feature extraction, they lack the temporal processing capabilities required for sequential IoMT data. In contrast, HCLR-IDS integrates LSTM and reinforcement learning to adapt to dynamic attack patterns over time, providing a comprehensive solution for real-time detection in IoMT environments. The LSTM model from Ref. (41), which reports 98.9% accuracy and high precision and recall, faces similar limitations. LSTM excels at processing sequential data but struggles with spatial feature extraction. HCLR-IDS addresses this limitation by incorporating CNN for spatial features, enhancing its overall performance. Furthermore, the Gradient Boosting Machine (GBM) model from Ref. (42) achieves 97.8% accuracy, but as a traditional machine learning technique, it lacks the capacity for adaptive learning in the dynamic IoMT setting. Finally, Ensemble Learning (RF + SVM) from Ref. (43), with 98.2% accuracy, demonstrates strong performance but does not adapt as quickly to new attack patterns compared to HCLR-IDS, which leverages reinforcement learning to continuously learn and improve its detection capabilities.

HCLR-IDS is specifically designed to address the challenges of real-time intrusion detection in IoMT healthcare environments, where high data volume, velocity, and evolving attack patterns can overwhelm traditional models. Its hybrid architecture, which combines deep learning for feature extraction and reinforcement learning for decision-making, allows it to capture both spatial and temporal features while adapting to new threats in real-time. CNN and LSTM excel at detecting complex attacks such as DDoS and spoofing, while DQN and PPO facilitate adaptive learning, enabling the system to improve detection accuracy over time. This results in low-latency processing, making HCLR-IDS particularly suitable for healthcare networks, where rapid response times are critical. The system integrates seamlessly with existing cybersecurity frameworks and can be deployed in distributed architectures across edge devices, ensuring scalability and real-time performance. By leveraging streaming data pipelines and hardware acceleration, HCLR-IDS efficiently processes high-throughput data, offering near-instantaneous detection even in large-scale hospital networks.

## 6 Conclusion

In conclusion, the proposed HCLR-IDS system demonstrates exceptional performance in securing IoMT healthcare networks. The model achieves a binary classification accuracy of 0.9958%, significantly outperforming traditional Intrusion Detection Systems (IDS). This high accuracy showcases the system’s ability to effectively identify malicious activities and distinguish them from normal network traffic, which is crucial for real-time protection in sensitive healthcare environments. Additionally, HCLR-IDS excels in multi-class classification, attaining an accuracy of 0.7773% across 18 distinct attack classes. This result highlights the system’s capability to detect a wide range of evolving and complex attack patterns, making it adaptable to the dynamic threats faced by IoMT systems. Its performance in handling both binary and multi-class classification reinforces its versatility in addressing various intrusion scenarios, further validating its effectiveness in providing comprehensive protection for healthcare IoT systems.

Future work will focus on enhancing the model’s ability to detect zero-day attacks and exploring advanced techniques such as attention mechanisms and ensemble methods to improve detection accuracy and the model’s adaptability to new attack strategies.

## Data Availability

The datasets presented in this study can be found in online repositories. The names of the repository/repositories and accession number(s) can be found in the article/supplementary material.
